# Statement of Retraction: MYCN contributes to the sensitization of acute myelogenous leukemia cells to cisplatin by targeting SRY-box transcription factor 4

**DOI:** 10.1080/21655979.2024.2340165

**Published:** 2024-04-19

We, the Publisher of the journal *Bioengineered*, have retracted the following article:

Xianbao Huang, Ling Qi, Wei Lu, Ziye Li, Wuping Li & Fei Li (2024) MYCN contributes to the sensitization of acute myelogenous leukemia cells to cisplatin by targeting SRY-box transcription factor 4, Bioengineered, 15:1, DOI: 10.1080/21655979.2021.1997697

Following publication, the authors have raised significant concerns about the reliability of the data presented in the article. As the Editor and Publisher also have concerns about the integrity of the reported results, all parties have agreed to retract the article to ensure correction of the scholarly record.

We have been informed in our decision-making by our editorial policies and the COPE guidelines.

The retracted article will remain online to maintain the scholarly record, but it will be digitally watermarked on each page as ‘Retracted’.

